# Supplementary material for: COVID-19 during pregnancy and risk of pregnancy loss (miscarriage or stillbirth): a systematic review protocol
Source: BMJ Open. 2022 Oct 3;12(10):e065588. doi: 10.1136/bmjopen-2022-065588 (PMC9534774; doi:10.1136/bmjopen-2022-065588)
Supplement: Supplementary data [file bmjopen-2022-065588supp001.pdf]

Appendix 1: Prisma-P checklist

PRISMA-P (Preferred Reporting Items for Systematic review and Meta-Analysis Protocols) 2015 checklist: recommended items to address in a systematic review protocol*

Section and topic	Item No	Checklist item
**ADMINISTRATIVE INFORMATION**		
Title:		
Identification	1a	Identify the report as a protocol of a systematic review
Update	1b	If the protocol is for an update of a previous systematic review, identify as such
Registration	2	If registered, provide the name of the registry (such as PROSPERO) and registration number
Authors:		
Contact	3a	Provide name, institutional affiliation, e-mail address of all protocol authors; provide physical mailing address of corresponding author
Contributions	3b	Describe contributions of protocol authors and identify the guarantor of the review
Amendments	4	If the protocol represents an amendment of a previously completed or published protocol, identify as such and list changes; otherwise, state plan for documenting important protocol amendments
Support:		
Sources	5a	Indicate sources of financial or other support for the review
Sponsor	5b	Provide name for the review funder and/or sponsor
Role of sponsor or funder	5c	Describe roles of funder(s), sponsor(s), and/or institution(s), if any, in developing the protocol
**INTRODUCTION**		
Rationale	6	Describe the rationale for the review in the context of what is already known
Objectives	7	Provide an explicit statement of the question(s) the review will address with reference to participants, interventions, comparators, and outcomes (PICO)
**METHODS**		
Eligibility criteria	8	Specify the study characteristics (such as PICO, study design, setting, time frame) and report characteristics (such as years considered, language, publication status) to be used as criteria for eligibility for the review
Information sources	9	Describe all intended information sources (such as electronic databases, contact with study authors, trial registers or other grey literature sources) with planned dates of coverage

Search strategy	10	Present draft of search strategy to be used for at least one electronic database, including planned limits, such that it could be repeated
Study records:		
Data management	11a	Describe the mechanism(s) that will be used to manage records and data throughout the review
Selection process	11b	State the process that will be used for selecting studies (such as two independent reviewers) through each phase of the review (that is, screening, eligibility and inclusion in meta-analysis)
Data collection process	11c	Describe planned method of extracting data from reports (such as piloting forms, done independently, in duplicate), any processes for obtaining and confirming data from investigators
Data items	12	List and define all variables for which data will be sought (such as PICO items, funding sources), any pre-planned data assumptions and simplifications
Outcomes and prioritization	13	List and define all outcomes for which data will be sought, including prioritization of main and additional outcomes, with rationale
Risk of bias in individual studies	14	Describe anticipated methods for assessing risk of bias of individual studies, including whether this will be done at the outcome or study level, or both; state how this information will be used in data synthesis
Data synthesis	15a	Describe criteria under which study data will be quantitatively synthesised
	15b	If data are appropriate for quantitative synthesis, describe planned summary measures, methods of handling data and methods of combining data from studies, including any planned exploration of consistency (such as I^2, Kendall's τ)
	15c	Describe any proposed additional analyses (such as sensitivity or subgroup analyses, meta-regression)
	15d	If quantitative synthesis is not appropriate, describe the type of summary planned
Meta-bias(es)	16	Specify any planned assessment of meta-bias(es) (such as publication bias across studies, selective reporting within studies)
Confidence in cumulative evidence	17	Describe how the strength of the body of evidence will be assessed (such as GRADE)

*** It is strongly recommended that this checklist be read in conjunction with the PRISMA-P Explanation and Elaboration (cite when available) for important clarification on the items. Amendments to a review protocol should be tracked and dated. The copyright for PRISMA-P (including checklist) is held by the PRISMA-P Group and is distributed under a Creative Commons Attribution Licence 4.0.**

From: Shamseer L, Moher D, Clarke M, Ghersi D, Liberati A, Petticrew M, Shekelle P, Stewart L, PRISMA-P Group. Preferred reporting items for systematic review and meta-analysis protocols (PRISMA-P) 2015: elaboration and explanation. BMJ. 2015 Jan 2;349(jan02 1):g7647.

Appendix 2: NICE Quality appraisal checklist for quantitative studies reporting correlations and associations

Checklist items are worded so that 1 of 5 responses is possible: ++ Indicates that for that particular aspect of study design, the study has been designed or conducted in such a way as to minimise the risk of bias. + Indicates that either the answer to the checklist question is not clear from the way the study is reported, or that the study may not have addressed all potential sources of bias for that particular aspect of study design. − Should be reserved for those aspects of the study design in which significant sources of bias may persist. Not reported (NR) Should be reserved for those aspects in which the study under review fails to report how they have (or might have) been considered. Not applicable (NA) Should be reserved for those study design aspects that are not applicable given the study design under review (for example, allocation concealment would not be applicable for case–control studies).

**Study identification:** Include full citation details		
**Study design:**<br>• Refer to the glossary of study designs (appendix D) and the algorithm for classifying experimental and observational study designs (appendix E) to best describe the paper's underpinning study design		
**Assessed by:**		
**Section 1: Population**		
**1.1 Is the source population or source area well described?**	++	Comments:

• Was the country (e.g. developed or non-developed, type of health care system), setting (primary schools, community centres etc), location (urban, rural), population demographics etc adequately described?	+<br>−<br>NR<br>NA	
**1.2 Is the eligible population or area representative of the source population or area?**<br>• Was the recruitment of individuals, clusters or areas well defined (e.g. advertisement, birth register)?<br>• Was the eligible population representative of the source? Were important groups underrepresented?	++<br>+<br>−<br>NR<br>NA	Comments:
**1.3 Do the selected participants or areas represent the eligible population or area?**<br>• Was the method of selection of participants from the eligible population well described?<br>• What % of selected individuals or clusters agreed to participate? Were there any sources of bias?	++<br>+<br>−<br>NR<br>NA	Comments:

• Were the inclusion or exclusion criteria explicit and appropriate?		
**Section 2: Method of selection of exposure (or comparison) group**		
**2.1 Selection of exposure (and comparison) group. How was selection bias minimised?**<br>• How was selection bias minimised?	++<br>+<br>−<br>NR<br>NA	Comments:
**2.2 Was the selection of explanatory variables based on a sound theoretical basis?**<br>• How sound was the theoretical basis for selecting the explanatory variables?	++<br>+<br>−<br>NR<br>NA	Comments:
**2.3 How well were likely confounding factors identified and controlled?**	++	Comments:

• Were there likely to be other confounding factors not considered or appropriately adjusted for?<br>• Was this sufficient to cause important bias?	+<br>−<br>NR<br>NA	
**2.4 Is the setting applicable to the UK?**<br>• Did the setting differ significantly from the UK?	++<br>+<br>−<br>NR<br>NA	Comments:
**Section 3: Outcomes**		
**3.1 Were the outcome measures and procedures reliable?**<br>• Were outcome measures subjective or objective (e.g. biochemically validated nicotine levels ++ vs self-reported smoking −)?	++<br>+<br>−	Comments:

• How reliable were outcome measures (e.g. inter- or intra-rater reliability scores)?<br>• Was there any indication that measures had been validated (e.g. validated against a gold standard measure or assessed for content validity)?	NR<br>NA	
**3.2 Were the outcome measurements complete?**<br>• Were all or most of the study participants who met the defined study outcome definitions likely to have been identified?	++<br>+<br>−<br>NR<br>NA	Comments:
**3.3 Were all the important outcomes assessed?**<br>• Were all the important benefits and harms assessed?<br>• Was it possible to determine the overall balance of benefits and harms of the intervention versus comparison?	++<br>+<br>−<br>NR<br>NA	Comments:

**3.4 Was there a similar follow-up time in exposure and comparison groups?**<br>• If groups are followed for different lengths of time, then more events are likely to occur in the group followed-up for longer distorting the comparison.<br>• Analyses can be adjusted to allow for differences in length of follow-up (e.g. using person-years).	++<br>+<br>−<br>NR<br>NA	Comments:
**3.5 Was follow-up time meaningful?**<br>• Was follow-up long enough to assess long-term benefits and harms?<br>• Was it too long, e.g. participants lost to follow-up?	++<br>+<br>−<br>NR<br>NA	Comments:
**Section 4: Analyses**		
**4.1 Was the study sufficiently powered to detect an intervention effect (if one exists)?**	++<br>+	Comments:

• A power of 0.8 (i.e. it is likely to see an effect of a given size if one exists, 80% of the time) is the conventionally accepted standard.<br>• Is a power calculation presented? If not, what is the expected effect size? Is the sample size adequate?	–<br>NR<br>NA	
**4.2 Were multiple explanatory variables considered in the analyses?**<br>• Were there sufficient explanatory variables considered in the analysis?	++<br>+<br>–<br>NR<br>NA	Comments:
**4.3 Were the analytical methods appropriate?**<br>• Were important differences in follow-up time and likely confounders adjusted for?	++<br>+<br>–<br>NR<br>NA	Comments:

**4.6 Was the precision of association given or calculable? Is association meaningful?**<br>• Were confidence intervals or p values for effect estimates given or possible to calculate?<br>• Were CIs wide or were they sufficiently precise to aid decision-making? If precision is lacking, is this because the study is under-powered?	++<br>+<br>−<br>NR<br>NA	Comments:
**Section 5: Summary**		
**5.1 Are the study results internally valid (i.e. unbiased)?**<br>• How well did the study minimise sources of bias (i.e. adjusting for potential confounders)?<br>• Were there significant flaws in the study design?	++<br>+<br>−	Comments:
**5.2 Are the findings generalisable to the source population (i.e. externally valid)?**<br>• Are there sufficient details given about the study to determine if the findings are generalisable to the source population?	++<br>+<br>−	Comments:

• Consider: participants, interventions and comparisons, outcomes, resource and policy implications.		
