# Supplementary material for: COVID-19 during pregnancy and risk of pregnancy loss (miscarriage or stillbirth): a systematic review protocol
Source: BMJ Open. 2022 Oct 3;12(10):e065588. doi: 10.1136/bmjopen-2022-065588 (PMC9534774; doi:10.1136/bmjopen-2022-065588)
Supplement: Author's manuscript [file bmjopen-2022-065588.draft_revisions.pdf]

BMJ Open

BMJ Open is committed to open peer review. As part of this commitment we make the peer review history of every article we publish publicly available.

When an article is published we post the peer reviewers' comments and the authors' responses online. We also post the versions of the paper that were used during peer review. These are the versions that the peer review comments apply to.

The versions of the paper that follow are the versions that were submitted during the peer review process. They are not the versions of record or the final published versions. They should not be cited or distributed as the published version of this manuscript.

BMJ Open is an open access journal and the full, final, typeset and author-corrected version of record of the manuscript is available on our site with no access controls, subscription charges or pay-per-view fees (http://bmjopen.bmj.com).

If you have any questions on BMJ Open's open peer review process please email info.bmjopen@bmj.com

BMJ Open

# Covid-19 during pregnancy and risk of pregnancy loss (miscarriage or stillbirth): a systematic review protocol

| | |
|---|---|
| Journal: | *BMJ Open* |
| Manuscript ID | bmjopen-2022-065588 |
| Article Type: | Protocol |
| Date Submitted by the Author: | 10-Jun-2022 |
| Complete List of Authors: | Campbell, Jennifer; London School of Hygiene and Tropical Medicine Faculty of Epidemiology and Population Health, NCDE; Medicines and Healthcare Products Regulatory Agency, Clinical Practice Research Datalink<br>Williams, Rachael; Medicines and Healthcare Products Regulatory Agency, Clinical Practice Research Datalink<br>Harley, Mia; London School of Hygiene and Tropical Medicine Faculty of Epidemiology and Population Health<br>Bhaskaran, Krishnan; London School of Hygiene and Tropical Medicine Faculty of Epidemiology and Population Health, NCDE |
| Keywords: | COVID-19, EPIDEMIOLOGY, OBSTETRICS |
| | |

SCHOLARONE™
Manuscripts

# Covid-19 during pregnancy and risk of pregnancy loss (miscarriage or stillbirth): a systematic review protocol

Jennifer Campbell [1 & 2] (jennifer.campbell@mhra.gov.uk), , Rachael Williams [2] (rachael.williams@mhra.gov.uk), Mia Harley [1] (mia.harley@lshtm.ac.uk), Krishnan Bhaskaran [1] (krishnan.bhaskaran@lshtm.ac.uk)

1. *London School of Hygiene and Tropical Medicine (LSHTM)*
2. *Clinical Practice Research Datalink (CPRD)*

Correspondence to Jennifer Campbell; jennifer.campbell@mhra.gov.uk

Clinical Practice Research Datalink, Medicines and Healthcare Products Regulatory Agency, 10 South Colonnade, Canary Wharf, London, E14 4PU, United Kingdom

For peer review only

**Keywords**

Systematic Review, COVID-19, SARS-CoV-2, Spontaneous Abortion, Stillbirth

**Word Count**: 1,440

**Abstract**

*Introduction*

*The Covid-19 pandemic has led to concerns about potential adverse pregnancy outcomes associated with infection, resulting in intensive research. Numerous studies have attempted to examine whether Covid-19 is associated with an increased risk of pregnancy loss. However, studies and reviews to date have drawn differing conclusions. The aim of this systematic review is to provide a summary of all quantitative research on the relationship between pregnancy loss and Covid-19 infection and if appropriate, to synthesise the evidence into an overall effect estimate.*

*Methods and Analysis*

*Three publication databases (Embase, PubMed and Cochrane) and four pre-print databases (MedRxiv, Lancet Preprint, Gates Open Research and Wellcome Open Research) will be searched. Boolean logic will be used to combine terms associated with pregnancy loss and Covid-19. The population of interest are pregnant women. Retrieved results will be assessed in two phases: 1. Abstract screening 2. Full text evaluation. All studies which compare pregnancy loss outcomes in woman who had Covid-19 versus those who did not quantitatively will be included. Narrative and none-English studies will be excluded. Two reviewers will screen independently, with results compared and discrepancies resolved by the study team. Study quality and risk of bias will be assessed using a quality appraisal tool. Results will be summarised descriptively and where possible synthesised in a meta-analysis.*

*Ethics and Dissemination*

*This systematic review requires no ethical approval. This review will be published in a peer-reviewed journal and provide an important update in a rapidly evolving field of research.*

*Registration: PROSPERO* CRD42022327437(1)



For peer review only - http://bmjopen.bmj.com/site/about/guidelines.xhtml

**Strengths and Limitations of this study**

- This Systematic Review will include both published and pre-print studies in an attempt to capture the very latest data and minimise publication bias.
- Study selection, data extraction and quality assessment will be performed independently by two researchers, which will ensure that all relevant studies are included without personal biases.
- All included studies will be assessed for quality using the NICE Quality appraisal checklist for quantitative studies reporting correlations and associations
- Studies which are not published in English will not be included. This limitation may cause language bias.

## Background

SARS-CoV-2 emerged as a new coronavirus at the end of 2019 spreading rapidly to cause a global pandemic of its associated illness Covid-19. Many millions of people around the world have been infected with the virus including pregnant women. However, due to the novelty of Covid-19 little is known about its potential effect on the unborn foetus and pregnancy outcomes. Aetiological hypotheses have been proposed as to ways in which Covid-19 may adversely affect pregnancy outcomes including potential increased risk of loss medicated by placental damage (2). Covid-19 in pregnancy has therefore been the subject of intense research and there have been numerous studies which have examined any potential adverse effect leading to reviews which have attempted to summarise the evidence (3–5).

As both the virus itself and our knowledge of its effects are constantly evolving both studies and reviews to date have drawn differing conclusions. Some have concluded an increased risk of pregnancy loss associated with Covid-19 infection (3,6–9) whilst others have concluded no increased risk (10–13). It is therefore important that we continue to review all emerging evidence in order to provide a full and current picture of any potential adverse risk.

The overall aim of this study is to identify and summarise all studies to date which have quantitatively compared pregnancy loss outcomes in women who contracted Covid-19 whilst pregnant versus those who did not. Where possible, quantitative estimates of associations between Covid-19 and pregnancy loss will be synthesised into an overall effect estimate.

## Methods

### *Study registration*

This protocol is prepared in accordance with the Preferred Reporting Item for Systematic Review and Meta-analysis (PRISMA-P) statement (Appendix 1) (14). This protocol is registered on the International Prospective Register of Systematic Reviews (PROSPERO) registration number CRD42022327437.

### *Eligibility Criteria*

The review will include all studies which have attempted to quantitatively assess the potential association between having Covid-19 during pregnancy and pregnancy loss.



For peer review only - http://bmjopen.bmj.com/site/about/guidelines.xhtml

The population of interest are pregnant women at any maternal age or gestation of pregnancy. The exposure of interest will be Covid-19 during pregnancy. The comparator population will be women who did not have Covid-19 during pregnancy. The outcome of interest will be pregnancy loss (miscarriage or stillbirth).

Table 1 gives the inclusion and exclusion criteria will be applied to identified studies.

**Table 1: Inclusion and Exclusion criteria**

| Inclusion | Exclusion |
|---|---|
| - Epidemiological studies which attempt to quantitatively assess any association between pregnancy loss and Covid-19 | -Non-English language publications including those where the summary is in English but not the full text.<br><br>- Narrative review articles, guidelines, editorials, or comments.<br><br>- Studies without a control or comparison group e.g. case reports<br><br>-Conference presentations |

*Information Sources*

Publication Databases to be searched: Embase (Ovid), PubMed, Cochrane.

Preprint platforms to be searched: MedRxiv, Lancet Preprint, Gates Open Research, Wellcome Open Research

*Search Strategy*

Search terms listed in Table 2 will be applied in the respective databases. Terms related to pregnancy loss will be combined with terms related to Covid-19 using AND logic. Only publications after the 1st of March 2020 will be searched

**Table 2: Database Search Strategy**

| Database | Miscarriage/Stillbirth | Covid-19 |
|---|---|---|
| Pubmed | "Abortion, Spontaneous" [MeSH] OR "Fetal Death" [MeSH] OR "Stillbirth" [MeSH] OR (miscarriage[MeSH Terms])) OR (miscarriages[MeSH Terms] OR Miscarriage* OR pregnancy loss* OR | "coronavirus"[MeSH] OR "coronavirus infections"[MeSH Terms] OR "coronavirus"[All Fields] OR "covid 2019"[All Fields] OR "SARS2"[All Fields] OR "SARS-CoV-2"[All Fields] OR "SARS-CoV-19"[All Fields] OR "severe acute respiratory syndrome coronavirus |



For peer review only - http://bmjopen.bmj.com/site/about/guidelines.xhtml

| | | |
|---|---|---|
| | spontaneous abortion* OR fetal loss* OR foetal loss* OR foetal death* OR fetal death* | 2" [supplementary concept] OR “coronavirus infection”[All Fields] OR "severe acute respiratory pneumonia outbreak”[All Fields] OR "novel cov"[All Fields] OR “2019ncov”[All Fields] OR “sars cov2”[All Fields] OR “cov22”[All Fields] OR “ncov”[All Fields] OR “covid19”[All Fields] OR “covid 19”[All Fields] OR “covid-19”[All Fields] OR “coronaviridae”[All Fields] OR "corona virus"[All Fields] |
| Embase | spontaneous abortion/exp OR stillbirth/exp OR stillbirth.m.p OR pregnancy loss/exp OR pregnancy loss.mp OR foetal death.m.p OR fetus death OR fetus death/exp NOT [medline]/lim | 'coronavirinae'/exp OR 'coronavirinae' OR 'coronaviridae infection'/exp OR 'coronaviridae infection' OR 'coronavirus disease 2019'/exp OR 'coronavirus'/exp OR coronavirus OR 'coronavirus infection'/de NOT [medline]/lim |
| Cochrane | Search for “stillbirth” OR “miscarriage” OR “foetal death rates” OR ““foetal death rate” OR “fetal death” OR “fetal death rate” OR “pregnancy loss rate” OR “pregnancy loss-rate” OR pregnancy “loss-rates” | Search for “coronavirus” in the Title Abstract Keyword fields |

To further increase the sensitivity of our search, the list of references from review articles relating to Covid-19 and pregnancy loss will be screened manually to identify other potentially eligible articles.

Due to the fast-moving nature of Covid-19 research we will also search databases of pre-print articles (15). The MedRxiv database will be searched via EMBASE using the search terms detailed above. The Lancet Preprint database will be searched for Obstetrics and Gynaecology articles which contain the term “Covid-19”. Gates Open Research and Wellcome Open Research will also be searched for “Covid-19” and “Pregnancy”. Pre-print databases were selected from a systematic examination of preprint platforms by Kirkam et al (16). Preprint articles will be flagged as such in any presentation of results.



For peer review only - http://bmjopen.bmj.com/site/about/guidelines.xhtml
For peer review only

*Data management and Selection Process*

Searches will be performed across all databases by reviewer 1. Records of the search terms, results from the search and the date of last run will be saved. Results will be exported into Mendeley where any duplicates results will be removed (17). Each article will be given a study id. The remaining articles will be screened for eligibility based on titles and abstracts by two independent reviewers applying the inclusion/exclusion criteria described above. Discrepancies will be discussed and where necessary will be discussed and decided by the whole study team. Full text articles will be obtained for all articles deemed eligible for inclusion from the initial screening. Articles will be divided and assessed independently by two reviewers after which the final selection will be agreed. Any reasons for exclusion will be recorded.

For peer review only



For peer review only - http://bmjopen.bmj.com/site/about/guidelines.xhtml

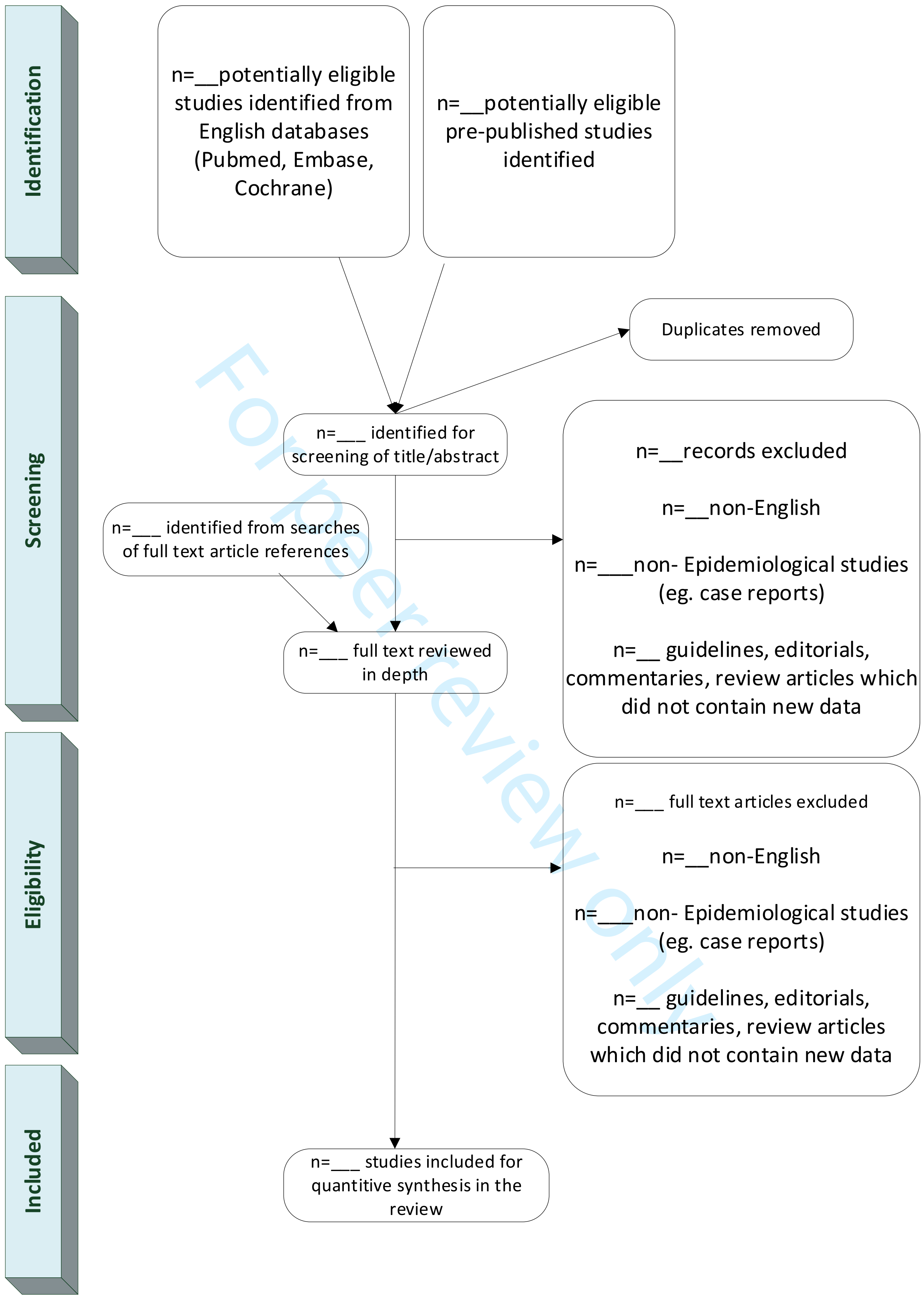


For peer review only



For peer review only - http://bmjopen.bmj.com/site/about/guidelines.xhtml

*Data Collection Process*

The example Data Capture table below will be pilot tested on a random sample of 5 included studies and revised if necessary. The finalised data capture form will then be completed by reviewers 1 & 2 independently for a sample of 10 studies to check concordance, after which each study will be examined by one reviewer.

| Study id | First Author, Year | Study Design | Location | Exposure Definition | Outcome Definition | N subjects | N Exposed | N miscarriage among the exposed | N Stillbirth among the exposed | N miscarriage among the unexposed | N Stillbirth among the unexposed | Statistical Measure and result reported in the paper | Was the study pre or post vaccine rollout? |
|---|---|---|---|---|---|---|---|---|---|---|---|---|---|
| | | | | | | | | | | | | | |

peer review only



For peer review only - http://bmjopen.bmj.com/site/about/guidelines.xhtml

*Assessment of Study Quality*

All included studies will be assessed for bias by reviewers using an adapted version of the NICE Quality appraisal checklist for quantitative studies reporting correlations and associations (Appendix 2)(18). This will be done in an Excel format to allow for easy compilation of responses. Decisions will be discussed any discrepancies resolved. Each study will then be awarded an overall study quality grade for external and internal validity from one of the three categories below which are based on the checklist criteria (Appendix 1).

- ++ All or most of the checklist criteria have been fulfilled, where they have not been fulfilled the conclusions are very unlikely to alter.
- + Some of the checklist criteria have been fulfilled, where they have not been fulfilled, or not adequately described, the conclusions are unlikely to alter.
- – Few or no checklist criteria have been fulfilled and the conclusions are likely or very likely to alter.

Studies deemed to be low quality (category -) will be excluded from any meta-analysis.

*Data Synthesis*

We will use Higgins and Thompson's I- squared statistic to quantify heterogeneity and if $I^2$ is >50% meta-analysis will be conducted in Stata using a Random-effects model (19). Where meta-analysis is attempted funnel plots will be used to assess publication bias (20). Where statistical pooling is not possible, findings will be presented in narrative form using tables to aid in data presentation. If possible, we will conduct subgroup analyses of studies reporting miscarriage and stillbirth separately. We will also look at any potential impact of the widespread use of Covid-19 vaccines by grouping studies into those conducted pre-and post-vaccine role out if possible.

*Patient and Public Involvement*

There will be no patient of public involvement in this project.

## Discussion

The Covid-19 pandemic has been a challenging time for pregnant women, knowledge on the potential risks of infection to them and their unborn babies is ever evolving. With Covid-19 now circulating widely in many countries and limited risk reduction measures in place it is important to try and fully understand the risks so that pregnant women can be advised appropriately. Reviews and studies to date on whether Covid-19 increases the risk of pregnancy loss have drawn mixed conclusions (3–5,9,13). Covid-19 research is a fast-moving area therefore it is important that reviews are regularly updated. This systematic review aims to provide a comprehensive overview of the latest evidence.



For peer review only - http://bmjopen.bmj.com/site/about/guidelines.xhtml

Covid-19 research moves very quickly, and preprint literature has become a key outlet for new research with many researchers opting to make their work available as quickly as possible. Including pre-publications in this review, something which previous reviews have not done, will allow us to obtain as current a picture as possible of all of the evidence. Inclusion of pre-print literature may also help to mitigate any risk of publication bias.

Vaccination against Covid-19 became widely available globally in 2021(21). In the UK pregnant women have been routinely advised to receive COVID-19 vaccination together with the rest of the population, according to their age and underlying health conditions since 16 April 2021(22). The widespread introduction of Covid-19 may have led to a decrease in potential risk or pregnancy loss. We hope to identify enough studies to allow us to examine separately those which were conducted before and after the vaccination roll out in order to provide an insight into any impact the vaccine may have had.

The results of this review can be used to inform public health messaging for pregnant women around the potential risks of Covid-19 infection. This research will also help to inform any future research studies planned on this question.

For peer review only

## Funding

This work is funded by Wellcome (Senior Research Fellowship for KB, grant number 220283/Z/20/Z). This work forms part of JC's PhD which is funded by The Clinical Practice Research Datalink, a division of the UK Medicines and Healthcare Products Regulatory Agency (Grant number N/A).

## Author Contributions

This protocol was written by JC with KB, RW and MH performing critical review. JC will act as the guarantor of the review.

## Competing Interests

The authors have no competing interests to declare

**Patient and Public Involvement**

Not applicable

**Ethics approval**

Not applicable

**Consent for publication**

Not applicable

**Availability of data**

Not applicable

**Acknowledgments**

Not applicable



For peer review only - http://bmjopen.bmj.com/site/about/guidelines.xhtml

## References


1. Campbell J, Harley M, Williams R, Bhaskaran K. Covid-19 during pregnancy and risk of pregnancy loss (miscarriage or stillbirth): a systematic review. PROSPERO 2022 CRD42022327437 Title [Internet]. Available from: https://www.crd.york.ac.uk/prospero/display_record.php?ID=CRD42022327437

2. Arthurs AL, Jankovic-Karasoulos T, Roberts CT. COVID-19 in pregnancy: What we know from the first year of the pandemic. Biochim Biophys Acta - Mol Basis Dis. 2021;1867(12).

3. Kazemi SN, Hajikhani B, Didar H, Hosseini SS, Haddadi S, Khalili F, et al. COVID-19 and cause of pregnancy loss during the pandemic: A systematic review. PLoS One [Internet]. 2021;16(8 August):1–10. Available from: http://dx.doi.org/10.1371/journal.pone.0255994

4. Soheili M, Ghobad M, Hamid Reza B, Soheili M, Mahidi Makhtari M, Moradi Y. Clinical manifestation and maternal complications and neonatal outcomes in pregnant women with COVID-19: a comprehensive evidence synthesis and meta-analysis. J Matern Fetal Neonatal Med. 2021;18:1–14.

5. Juan J, Gil MM, Rong Z, Zhang Y, Yang H, Poon LC. Effect of coronavirus disease 2019 (COVID-19) on maternal, perinatal and neonatal outcome: systematic review. Ultrasound Obstet Gynecol. 2020;56(1):15–27.

6. Hazari KS, Abdeldayem R, Paulose L, Kurien N, Almahloul Z, Mohammad H, et al. Covid-19 infection in pregnant women in Dubai: a case-control study. BMC Pregnancy Childbirth [Internet]. 2021;21(1):1–10. Available from: https://doi.org/10.1186/s12884-021-04130-8

7. González Rodríguez L, Oreja Cuesta AB, Pardo Pumar MI, Ferriols-Pérez E, Pedró Carulla R, Bernardo Vega R, et al. SARS-CoV-2 infection in early first-trimester miscarriages: a prospective observational study. Reprod Biomed Online [Internet]. 2022;44(1):127–30. Available from: https://doi.org/10.1016/j.rbmo.2021.09.010

8. Webster WS, Freeman JAD. Prescription drugs and pregnancy. Expert Opin Pharmacother. 2003 Jun;4(6):949–61.

9. Pathirathna ML, Samarasekara BPP, Dasanayake TS, Saravanakumar P, Weerasekara I. Adverse Perinatal Outcomes in COVID-19 Infected Pregnant Women: A Systematic Review and Meta-Analysis. Healthc. 2022;10(2).

10. Sacinti KG, Kalafat E, Sukur YE, Koc A. Increased incidence of first-trimester miscarriage during the COVID-19 pandemic. Ultrasound Obstet Gynecol. 2021;57(6):1013–4.

11. Francesco D'Antonio, MD; Cihat Sen MDDM. Maternal and perinatal outcomes in high compared to working group on coronavirus disease 2019. 2020;(January).

12. Cosma S, Carosso AR, Cusato J, Borella F, Carosso M, Bovetti M, et al. Coronavirus disease 2019 and first-trimester spontaneous abortion: a case-control study of 225 pregnant patients. Am J Obstet Gynecol [Internet]. 2021;224(4):391.e1-391.e7.




For peer review only - http://bmjopen.bmj.com/site/about/guidelines.xhtml

Available from: https://doi.org/10.1016/j.ajog.2020.10.005

13. Cavalcante MB, de Melo Bezerra Cavalcante CT, Cavalcante ANM, Sarno M, Barini R, Kwak-Kim J. COVID-19 and miscarriage: From immunopathological mechanisms to actual clinical evidence. J Reprod Immunol [Internet]. 2021;148(September):103382. Available from: https://doi.org/10.1016/j.jri.2021.103382

14. Shamseer L, Moher D, Clarke M, Ghersi D, Liberati A, Petticrew M, et al. Preferred reporting items for systematic review and meta-analysis protocols (prisma-p) 2015: Elaboration and explanation. BMJ [Internet]. 2015;349(January):1–25. Available from: http://dx.doi.org/doi:10.1136/bmj.g7647

15. Clyne B, Walsh KA, O'Murchu E, Sharp MK, Comber L, O' Brien KK, et al. Using preprints in evidence synthesis: Commentary on experience during the COVID-19 pandemic. J Clin Epidemiol [Internet]. 2021;138:203–10. Available from: https://doi.org/10.1016/j.jclinepi.2021.05.010

16. Kirkham JJ, Penfold NC, Murphy F, Boutron I, Ioannidis JP, Polka J, et al. Systematic examination of preprint platforms for use in the medical and biomedical sciences setting. BMJ Open. 2020;10(12):1–8.

17. Mendeley Ltd. Mendeley Reference Manager [Internet]. 2020. Available from: https://www.mendeley.com

18. NICE. Methods for the development of NICE public health guidance (third edition ). Natl Inst Heal Clin Excell [Internet]. 2012;(September):284. Available from: https://www.nice.org.uk/process/pmg4/resources/methods-for-the-development-of-nice-public-health-guidance-third-edition-pdf-2007967445701

19. StataCorp. Stata Statistical Software: Release 17. College Station, TX: StataCorp LLC.; 2021.

20. Egger M, Smith GD, Schneider M, Minder C. Bias in meta-analysis detected by a simple, graphical test. Br Med J. 1997;315(7109):629–34.

21. Ritchie H, Mathieu E, Rodés-Guirao L, Appel C, Giattino C, Ortiz-Ospina E, et al. Coronavirus Pandemic (COVID-19) [Internet]. Published online at OurWorldInData.org. 2020. Available from: https://ourworldindata.org/coronavirus

22. UK Health Security Agency. COVID-19: The Green Book chapter 14a. In: The Green Book [Internet]. 2022. Available from: https://www.gov.uk/government/publications/covid-19-the-green-book-chapter-14a%0A

For peer review only

Appendix 1: Prisma-P checklist

**PRISMA-P (Preferred Reporting Items for Systematic review and Meta-Analysis Protocols) 2015 checklist: recommended items to address in a systematic review protocol***

| Section and topic | Item No | Checklist item |
|---|---|---|
| **ADMINISTRATIVE INFORMATION** | | |
| Title: | | |
| Identification | 1a | Identify the report as a protocol of a systematic review |
| Update | 1b | If the protocol is for an update of a previous systematic review, identify as such |
| Registration | 2 | If registered, provide the name of the registry (such as PROSPERO) and registration number |
| Authors: | | |
| Contact | 3a | Provide name, institutional affiliation, e-mail address of all protocol authors; provide physical mailing address of corresponding author |
| Contributions | 3b | Describe contributions of protocol authors and identify the guarantor of the review |
| Amendments | 4 | If the protocol represents an amendment of a previously completed or published protocol, identify as such and list changes; otherwise, state plan for documenting important protocol amendments |
| Support: | | |
| Sources | 5a | Indicate sources of financial or other support for the review |
| Sponsor | 5b | Provide name for the review funder and/or sponsor |
| Role of sponsor or funder | 5c | Describe roles of funder(s), sponsor(s), and/or institution(s), if any, in developing the protocol |
| **INTRODUCTION** | | |
| Rationale | 6 | Describe the rationale for the review in the context of what is already known |
| Objectives | 7 | Provide an explicit statement of the question(s) the review will address with reference to participants, interventions, comparators, and outcomes (PICO) |
| **METHODS** | | |
| Eligibility criteria | 8 | Specify the study characteristics (such as PICO, study design, setting, time frame) and report characteristics (such as years considered, language, publication status) to be used as criteria for eligibility for the review |
| Information sources | 9 | Describe all intended information sources (such as electronic databases, contact with study authors, trial registers or other grey literature sources) with planned dates of coverage |

For peer review only

| | | |
|---|---|---|
| Search strategy | 10 | Present draft of search strategy to be used for at least one electronic database, including planned limits, such that it could be repeated |
| Study records: | | |
| Data management | 11a | Describe the mechanism(s) that will be used to manage records and data throughout the review |
| Selection process | 11b | State the process that will be used for selecting studies (such as two independent reviewers) through each phase of the review (that is, screening, eligibility and inclusion in meta-analysis) |
| Data collection process | 11c | Describe planned method of extracting data from reports (such as piloting forms, done independently, in duplicate), any processes for obtaining and confirming data from investigators |
| Data items | 12 | List and define all variables for which data will be sought (such as PICO items, funding sources), any pre-planned data assumptions and simplifications |
| Outcomes and prioritization | 13 | List and define all outcomes for which data will be sought, including prioritization of main and additional outcomes, with rationale |
| Risk of bias in individual studies | 14 | Describe anticipated methods for assessing risk of bias of individual studies, including whether this will be done at the outcome or study level, or both; state how this information will be used in data synthesis |
| Data synthesis | 15a | Describe criteria under which study data will be quantitatively synthesised |
| | 15b | If data are appropriate for quantitative synthesis, describe planned summary measures, methods of handling data and methods of combining data from studies, including any planned exploration of consistency (such as $I^2$, Kendall's $\tau$) |
| | 15c | Describe any proposed additional analyses (such as sensitivity or subgroup analyses, meta-regression) |
| | 15d | If quantitative synthesis is not appropriate, describe the type of summary planned |
| Meta-bias(es) | 16 | Specify any planned assessment of meta-bias(es) (such as publication bias across studies, selective reporting within studies) |
| Confidence in cumulative evidence | 17 | Describe how the strength of the body of evidence will be assessed (such as GRADE) |

*** It is strongly recommended that this checklist be read in conjunction with the PRISMA-P Explanation and Elaboration (cite when available) for important clarification on the items. Amendments to a review protocol should be tracked and dated. The copyright for PRISMA-P (including checklist) is held by the PRISMA-P Group and is distributed under a Creative Commons Attribution Licence 4.0.**

*From: Shamseer L, Moher D, Clarke M, Ghersi D, Liberati A, Petticrew M, Shekelle P, Stewart L, PRISMA-P Group. Preferred reporting items for systematic review and meta-analysis protocols (PRISMA-P) 2015: elaboration and explanation. BMJ. 2015 Jan 2;349(jan02 1):g7647.*

For peer review only

Appendix 2: NICE Quality appraisal checklist for quantitative studies reporting correlations and associations

Checklist items are worded so that 1 of 5 responses is possible: ++ Indicates that for that particular aspect of study design, the study has been designed or conducted in such a way as to minimise the risk of bias. + Indicates that either the answer to the checklist question is not clear from the way the study is reported, or that the study may not have addressed all potential sources of bias for that particular aspect of study design. − Should be reserved for those aspects of the study design in which significant sources of bias may persist. Not reported (NR) Should be reserved for those aspects in which the study under review fails to report how they have (or might have) been considered. Not applicable (NA) Should be reserved for those study design aspects that are not applicable given the study design under review (for example, allocation concealment would not be applicable for case–control studies).

| | | |
|---|---|---|
| **Study identification:** Include full citation details | | |
| **Study design:**<br>• Refer to the glossary of study designs (appendix D) and the algorithm for classifying experimental and observational study designs (appendix E) to best describe the paper's underpinning study design | | |
| **Assessed by:** | | |
| **Section 1: Population** | | |
| **1.1 Is the source population or source area well described?** | ++ | Comments: |

| | | |
|---|---|---|
| • Was the country (e.g. developed or non-developed, type of health care system), setting (primary schools, community centres etc), location (urban, rural), population demographics etc adequately described? | +<br>–<br>NR<br>NA | |
| **1.2 Is the eligible population or area representative of the source population or area?**<br>• Was the recruitment of individuals, clusters or areas well defined (e.g. advertisement, birth register)?<br>• Was the eligible population representative of the source? Were important groups underrepresented? | ++<br>+<br>–<br>NR<br>NA | Comments: |
| **1.3 Do the selected participants or areas represent the eligible population or area?**<br>• Was the method of selection of participants from the eligible population well described?<br>• What % of selected individuals or clusters agreed to participate? Were there any sources of bias? | ++<br>+<br>–<br>NR<br>NA | Comments: |

| | | |
|---|---|---|
| • Were the inclusion or exclusion criteria explicit and appropriate? | | |
| **Section 2: Method of selection of exposure (or comparison) group** | | |
| **2.1 Selection of exposure (and comparison) group. How was selection bias minimised?**<br>• How was selection bias minimised? | ++<br>+<br>−<br>NR<br>NA | Comments: |
| **2.2 Was the selection of explanatory variables based on a sound theoretical basis?**<br>• How sound was the theoretical basis for selecting the explanatory variables? | ++<br>+<br>−<br>NR<br>NA | Comments: |
| **2.3 How well were likely confounding factors identified and controlled?** | ++ | Comments: |

| | | |
|---|---|---|
| • Were there likely to be other confounding factors not considered or appropriately adjusted for?<br>• Was this sufficient to cause important bias? | +<br>−<br>NR<br>NA | |
| **2.4 Is the setting applicable to the UK?**<br>• Did the setting differ significantly from the UK? | ++<br>+<br>−<br>NR<br>NA | Comments: |
| **Section 3: Outcomes** | | |
| **3.1 Were the outcome measures and procedures reliable?**<br>• Were outcome measures subjective or objective (e.g. biochemically validated nicotine levels ++ vs self-reported smoking −)? | ++<br>+<br>− | Comments: |

| | | |
|---|---|---|
| • How reliable were outcome measures (e.g. inter- or intra-rater reliability scores)?<br>• Was there any indication that measures had been validated (e.g. validated against a gold standard measure or assessed for content validity)? | NR<br>NA | |
| **3.2 Were the outcome measurements complete?**<br>• Were all or most of the study participants who met the defined study outcome definitions likely to have been identified? | ++<br>+<br>−<br>NR<br>NA | Comments: |
| **3.3 Were all the important outcomes assessed?**<br>• Were all the important benefits and harms assessed?<br>• Was it possible to determine the overall balance of benefits and harms of the intervention versus comparison? | ++<br>+<br>−<br>NR<br>NA | Comments: |

| | | |
|---|---|---|
| **3.4 Was there a similar follow-up time in exposure and comparison groups?**<br>• If groups are followed for different lengths of time, then more events are likely to occur in the group followed-up for longer distorting the comparison.<br>• Analyses can be adjusted to allow for differences in length of follow-up (e.g. using person-years). | ++<br>+<br>−<br>NR<br>NA | Comments: |
| **3.5 Was follow-up time meaningful?**<br>• Was follow-up long enough to assess long-term benefits and harms?<br>• Was it too long, e.g. participants lost to follow-up? | ++<br>+<br>−<br>NR<br>NA | Comments: |
| **Section 4: Analyses** | | |
| **4.1 Was the study sufficiently powered to detect an intervention effect (if one exists)?** | ++<br>+ | Comments: |

| | | |
|---|---|---|
| • A power of 0.8 (i.e. it is likely to see an effect of a given size if one exists, 80% of the time) is the conventionally accepted standard.<br>• Is a power calculation presented? If not, what is the expected effect size? Is the sample size adequate? | −<br>NR<br>NA | |
| **4.2 Were multiple explanatory variables considered in the analyses?**<br>• Were there sufficient explanatory variables considered in the analysis? | ++<br>+<br>−<br>NR<br>NA | Comments: |
| **4.3 Were the analytical methods appropriate?**<br>• Were important differences in follow-up time and likely confounders adjusted for? | ++<br>+<br>−<br>NR<br>NA | Comments: |

| | | |
|---|---|---|
| **4.6 Was the precision of association given or calculable? Is association meaningful?**<br>• Were confidence intervals or p values for effect estimates given or possible to calculate?<br>• Were CIs wide or were they sufficiently precise to aid decision-making? If precision is lacking, is this because the study is under-powered? | ++<br>+<br>−<br>NR<br>NA | Comments: |
| **Section 5: Summary** | | |
| **5.1 Are the study results internally valid (i.e. unbiased)?**<br>• How well did the study minimise sources of bias (i.e. adjusting for potential confounders)?<br>• Were there significant flaws in the study design? | ++<br>+<br>− | Comments: |
| **5.2 Are the findings generalisable to the source population (i.e. externally valid)?**<br>• Are there sufficient details given about the study to determine if the findings are generalisable to the source population? | ++<br>+<br>− | Comments: |

| | | |
|---|---|---|
| • Consider: participants, interventions and comparisons, outcomes, resource and policy implications. | | |

For peer review only

# Reporting checklist for protocol of a systematic review and meta analysis.

Based on the PRISMA-P guidelines.

## Instructions to authors

Complete this checklist by entering the page numbers from your manuscript where readers will find each of the items listed below.

Your article may not currently address all the items on the checklist. Please modify your text to include the missing information. If you are certain that an item does not apply, please write "n/a" and provide a short explanation.

Upload your completed checklist as an extra file when you submit to a journal.

In your methods section, say that you used the PRISMA-Preporting guidelines, and cite them as:

Moher D, Shamseer L, Clarke M, Ghersi D, Liberati A, Petticrew M, Shekelle P, Stewart LA. Preferred Reporting Items for Systematic Review and Meta-Analysis Protocols (PRISMA-P) 2015 statement. Syst Rev. 2015;4(1):1.

| | | Reporting Item | Page Number |
|---|---|---|---|
| **Title** | | | |
| Identification | #1a | Identify the report as a protocol of a systematic review | 1 |
| Update | #1b | If the protocol is for an update of a previous systematic review, identify as such | n/a |
| **Registration** | | | |
| | #2 | If registered, provide the name of the registry (such as PROSPERO) and registration number | 1 |
| **Authors** | | | |
| Contact | #3a | Provide name, institutional affiliation, e-mail address of all protocol authors; provide physical mailing address of corresponding author | 1 |
| Contribution | #3b | Describe contributions of protocol authors and identify the guarantor of the review | 8 |

For peer review only

| | | | |
|---|---|---|---|
| **Amendments** | | | |
| | #4 | If the protocol represents an amendment of a previously completed or published protocol, identify as such and list changes; otherwise, state plan for documenting important protocol amendments | n/a |
| **Support** | | | |
| Sources | #5a | Indicate sources of financial or other support for the review | 8 |
| Sponsor | #5b | Provide name for the review funder and / or sponsor | 8 |
| Role of sponsor or funder | #5c | Describe roles of funder(s), sponsor(s), and / or institution(s), if any, in developing the protocol | n/a |
| **Introduction** | | | |
| Rationale | #6 | Describe the rationale for the review in the context of what is already known | 2 |
| Objectives | #7 | Provide an explicit statement of the question(s) the review will address with reference to participants, interventions, comparators, and outcomes (PICO) | 2 |
| **Methods** | | | |
| Eligibility criteria | #8 | Specify the study characteristics (such as PICO, study design, setting, time frame) and report characteristics (such as years considered, language, publication status) to be used as criteria for eligibility for the review | 2-3 |
| Information sources | #9 | Describe all intended information sources (such as electronic databases, contact with study authors, trial registers or other grey literature sources) with planned dates of coverage | 3-4 |
| Search strategy | #10 | Present draft of search strategy to be used for at least one electronic database, including planned limits, such that it could be repeated | 3-4 |
| Study records - data management | #11a | Describe the mechanism(s) that will be used to manage records and data throughout the review | 4-5 |
| Study records - selection process | #11b | State the process that will be used for selecting studies (such as two independent reviewers) through each phase of the review (that is, screening, eligibility and inclusion in meta-analysis) | 5 |
| Study records - data | #11c | Describe planned method of extracting data from reports (such as | 6 |

For peer review only

| | | | |
|---|---|---|---|
| collection process | | piloting forms, done independently, in duplicate), any processes for obtaining and confirming data from investigators | |
| Data items | #12 | List and define all variables for which data will be sought (such as PICO items, funding sources), any pre-planned data assumptions and simplifications | 6 |
| Outcomes and prioritization | #13 | List and define all outcomes for which data will be sought, including prioritization of main and additional outcomes, with rationale | 2 |
| Risk of bias in individual studies | #14 | Describe anticipated methods for assessing risk of bias of individual studies, including whether this will be done at the outcome or study level, or both; state how this information will be used in data synthesis | 7 |
| Data synthesis | #15a | Describe criteria under which study data will be quantitatively synthesised | 7 |
| Data synthesis | #15b | If data are appropriate for quantitative synthesis, describe planned summary measures, methods of handling data and methods of combining data from studies, including any planned exploration of consistency (such as I2, Kendall's τ) | 7 |
| Data synthesis | #15c | Describe any proposed additional analyses (such as sensitivity or subgroup analyses, meta-regression) | 7 |
| Data synthesis | #15d | If quantitative synthesis is not appropriate, describe the type of summary planned | 7 |
| Meta-bias(es) | #16 | Specify any planned assessment of meta-bias(es) (such as publication bias across studies, selective reporting within studies) | 7 |
| Confidence in cumulative evidence | #17 | Describe how the strength of the body of evidence will be assessed (such as GRADE) | 7 |

The PRISMA-P elaboration and explanation paper is distributed under the terms of the Creative Commons Attribution License CC-BY. This checklist was completed on 09. June 2022 using https://www.goodreports.org/, a tool made by the EQUATOR Network in collaboration with Penelope.ai

For peer review only

BMJ Open

# Covid-19 during pregnancy and risk of pregnancy loss (miscarriage or stillbirth): a systematic review protocol

| | |
|---|---|
| Journal: | *BMJ Open* |
| Manuscript ID | bmjopen-2022-065588.R1 |
| Article Type: | Protocol |
| Date Submitted by the Author: | 16-Sep-2022 |
| Complete List of Authors: | Campbell, Jennifer; London School of Hygiene and Tropical Medicine Faculty of Epidemiology and Population Health, NCDE; Medicines and Healthcare Products Regulatory Agency, Clinical Practice Research Datalink<br>Williams, Rachael; Medicines and Healthcare Products Regulatory Agency, Clinical Practice Research Datalink<br>Harley, Mia; London School of Hygiene and Tropical Medicine Faculty of Epidemiology and Population Health<br>Bhaskaran, Krishnan; London School of Hygiene and Tropical Medicine Faculty of Epidemiology and Population Health, NCDE |
| <b>Primary Subject Heading</b>: | Epidemiology |
| Secondary Subject Heading: | Obstetrics and gynaecology, Infectious diseases |
| Keywords: | COVID-19, EPIDEMIOLOGY, OBSTETRICS |
| | |

SCHOLARONE™
Manuscripts

# Covid-19 during pregnancy and risk of pregnancy loss (miscarriage or stillbirth): a systematic review protocol

Jennifer Campbell [1 & 2] (jennifer.campbell@mhra.gov.uk), Rachael Williams [2] (rachael.williams@mhra.gov.uk), Mia Harley [1] (mia.harley@lshtm.ac.uk), Krishnan Bhaskaran [1] (krishnan.bhaskaran@lshtm.ac.uk)

1. *London School of Hygiene and Tropical Medicine (LSHTM)*
2. *Clinical Practice Research Datalink (CPRD)*

Correspondence to Jennifer Campbell; jennifer.campbell@mhra.gov.uk

Clinical Practice Research Datalink, Medicines and Healthcare Products Regulatory Agency, 10 South Colonnade, Canary Wharf, London, E14 4PU, United Kingdom

**Keywords**

Systematic Review, COVID-19, SARS-CoV-2, Spontaneous Abortion, Stillbirth

**Word Count**: 1,440

## Abstract

*Introduction*

*The Covid-19 pandemic has led to concerns about potential adverse pregnancy outcomes associated with infection, resulting in intensive research. Numerous studies have attempted to examine whether Covid-19 is associated with an increased risk of pregnancy loss. However, studies and reviews to date have drawn differing conclusions. The aim of this systematic review is to provide a summary of all quantitative research on the relationship between pregnancy loss and Covid-19 infection and if appropriate, to synthesise the evidence into an overall effect estimate.*

*Methods and Analysis*

*Three publication databases (Embase, PubMed and Cochrane) and four pre-print databases (MedRxiv, Lancet Preprint, Gates Open Research and Wellcome Open Research) will be searched. Boolean logic will be used to combine terms associated with pregnancy loss and Covid-19. The population of interest are pregnant women. Retrieved results will be assessed in two phases: 1. Abstract screening 2. Full text evaluation. All studies which compare pregnancy loss outcomes in woman who had Covid-19 versus those who did not quantitatively will be included. Narrative and non-English studies will be excluded. Two reviewers will screen independently, with results compared and discrepancies resolved by the study team. Study quality and risk of bias will be assessed using a quality appraisal tool. Results will be summarised descriptively and where possible synthesised in a meta-analysis.*

*Ethics and Dissemination*

*This systematic review requires no ethical approval. This review will be published in a peer-reviewed journal and provide an important update in a rapidly evolving field of research.*

*Registration: PROSPERO* CRD42022327437



For peer review only - http://bmjopen.bmj.com/site/about/guidelines.xhtml

## Strengths and Limitations of this study

- This Systematic Review will include both published and pre-print studies in an attempt to capture the very latest data and minimise publication bias.
- Study selection, data extraction and quality assessment will be performed independently by two researchers, which will ensure that all relevant studies are included without personal biases.
- All included studies will be assessed for quality using the NICE Quality appraisal checklist for quantitative studies reporting correlations and associations
- Studies which are not published in English will not be included. This limitation may cause language bias.

## Background

SARS-CoV-2 emerged as a new coronavirus at the end of 2019 spreading rapidly to cause a global pandemic of its associated illness Covid-19. Many millions of people around the world have been infected with the virus including pregnant women. However, due to the novelty of Covid-19 little is known about its potential effect on the unborn foetus and pregnancy outcomes. Aetiological hypotheses have been proposed as to ways in which Covid-19 may adversely affect pregnancy outcomes including potential increased risk of loss medicated by placental damage (1). Covid-19 in pregnancy has therefore been the subject of intense research and there have been numerous studies which have examined any potential adverse effect leading to reviews which have attempted to summarise the evidence (2–4).

As both the virus itself and our knowledge of its effects are constantly evolving both studies and reviews to date have drawn differing conclusions. Some have concluded an increased risk of pregnancy loss associated with Covid-19 infection (2,5–9) whilst others have concluded no increased risk (10–13).Many early reviews of this question included only case reports as no comparative studies were available (14–17). The latest published systematic review on this question by Pathirathna et al included studies published prior to June 2021 just over one year into the Covid-19 pandemic and like all reviews to date on this topic they noted the need for further research (8). Since this review there have been numerous additional studies published and there has been a global roll-out of vaccinations for Covid-19 to pregnant women. It is therefore important that we continue to review all emerging evidence in order to provide a full and current picture of any potential adverse risk.

The overall aim of this study is to identify and summarise all studies to date which have quantitatively compared pregnancy loss outcomes in women who contracted Covid-19 whilst pregnant versus those who did not. Where possible, quantitative estimates of associations between Covid-19 and pregnancy loss will be synthesised into an overall effect estimate.

## Methods

### *Study registration*

This protocol is prepared in accordance with the Preferred Reporting Item for Systematic Review and Meta-analysis (PRISMA-P) statement (Appendix 1) (18). This protocol is registered on the International Prospective Register of Systematic Reviews (PROSPERO) registration number CRD42022327437.



For peer review only - http://bmjopen.bmj.com/site/about/guidelines.xhtml

*Eligibility Criteria*

The review will include all studies which have attempted to quantitatively assess the potential association between having Covid-19 during pregnancy and pregnancy loss.

The population of interest are pregnant women at any maternal age or gestation of pregnancy. The exposure of interest will be Covid-19 during pregnancy. We will include all studies which attempt to ascertain Covid-19 exposure in pregnancy regardless of the method of diagnosis. The comparator population will be women who did not have Covid-19 during pregnancy. The outcome of interest will be pregnancy loss (miscarriage or stillbirth).

Table 1 gives the inclusion and exclusion criteria will be applied to identified studies.

**Table 1: Inclusion and Exclusion criteria**

| Inclusion | Exclusion |
|---|---|
| - Epidemiological studies which attempt to quantitatively assess any association between pregnancy loss and Covid-19 (Study designs may include prospective and retrospective cohort studies, case control studies and cross-sectional studies) | -Non-English language publications including those where the summary is in English but not the full text.<br><br>- Narrative review articles, guidelines, editorials, or comments.<br><br>- Studies without a control or comparison group e.g. case reports<br><br>-Conference presentations |

For peer review only

*Information Sources*

Publication Databases to be searched: Embase (Ovid), PubMed, Cochrane.

Preprint platforms to be searched: MedRxiv, Lancet Preprint, Gates Open Research, Wellcome Open Research

*Search Strategy*

Search terms listed in Table 2 will be applied in the respective databases. Terms related to pregnancy loss will be combined with terms related to Covid-19 using AND logic. Only publications after the 1st of March 2020 will be searched

**Table 2: Database Search Strategy**

| Database | Dates of search coverage | Miscarriage/Stillbirth | Covid-19 |
|---|---|---|---|
| Pubmed | 01/03/2020-<br>Current date | “Abortion, Spontaneous” [MeSH] OR “Fetal Death” [MeSH] OR “Stillbirth” [MeSH] OR | "coronavirus"[MeSH] OR "coronavirus infections"[MeSH Terms] OR "coronavirus"[All Fields] OR "covid 2019"[All |

| | | | |
|---|---|---|---|
| | | (miscarriage[MeSH Terms])) OR (miscarriages[MeSH Terms] OR Miscarriage* OR pregnancy loss* OR spontaneous abortion* OR fetal loss* OR foetal loss* OR foetal death* OR fetal death* | Fields] OR "SARS2"[All Fields] OR "SARS-CoV-2"[All Fields] OR "SARS-CoV-19"[All Fields] OR "severe acute respiratory syndrome coronavirus 2" [supplementary concept] OR “coronavirus infection”[All Fields] OR "severe acute respiratory pneumonia outbreak"[All Fields] OR "novel cov"[All Fields] OR “2019ncov”[All Fields] OR “sars cov2”[All Fields] OR “cov22”[All Fields] OR “ncov”[All Fields] OR “covid19”[All Fields] OR “covid 19”[All Fields] OR “covid-19”[All Fields] OR “coronaviridae”[All Fields] OR "corona virus"[All Fields] |
| Embase | 01/03/2020-Current date | spontaneous abortion/exp OR stillbirth/exp OR stillbirth.m.p OR pregnancy loss/exp OR pregnancy loss.mp OR foetal death.m.p OR fetus death OR fetus death/exp NOT [medline]/lim | 'coronavirinae'/exp OR 'coronavirinae' OR 'coronaviridae infection'/exp OR 'coronaviridae infection' OR 'coronavirus disease 2019'/exp OR 'coronavirus'/exp OR coronavirus OR 'coronavirus infection'/de NOT [medline]/lim |
| Cochrane | 01/03/2020-Current date | Search for “stillbirth” OR “miscarriage” OR “foetal death rates” OR ““foetal death rate” OR “fetal death” OR “fetal death rate” OR “pregnancy loss rate” OR “pregnancy loss-rate” OR pregnancy “loss-rates” | Search for “coronavirus” in the Title Abstract Keyword fields |



For peer review only - http://bmjopen.bmj.com/site/about/guidelines.xhtml

To further increase the sensitivity of our search, the list of references from review articles relating to Covid-19 and pregnancy loss will be screened manually to identify other potentially eligible articles.

Due to the fast-moving nature of Covid-19 research we will also search databases of pre-print articles (19). The MedRxiv database will be searched via EMBASE using the search terms detailed above. The Lancet Preprint database will be searched for Obstetrics and Gynaecology articles which contain the term "Covid-19". Gates Open Research and Wellcome Open Research will also be searched for "Covid-19" and "Pregnancy". Pre-print databases were selected from a systematic examination of preprint platforms by Kirkam et al (20). Preprint articles will be flagged as such in any presentation of results.

*Data management and Selection Process*

Searches will be performed across all databases by reviewer 1. Records of the search terms, results from the search and the date of last run will be saved. Results will be exported into Mendeley where any duplicates results will be removed (21). Each article will be given a study id. The remaining articles will be screened for eligibility based on titles and abstracts by two independent reviewers applying the inclusion/exclusion criteria described above. Discrepancies will be discussed and where necessary will be discussed and decided by the whole study team. Full text articles will be obtained for all articles deemed eligible for inclusion from the initial screening. Articles will be divided and assessed independently by two reviewers after which the final selection will be agreed. Any reasons for exclusion will be recorded.The study selection process is outlined in Figure 1.

**Figure 1: PRISMA Diagram of Study Selection Process**

.

For peer review only

*Data Collection Process*

The example Data Capture table below (Table 3) will be pilot tested on a random sample of 5 included studies and revised if necessary. The finalised data capture form will then be completed by reviewers 1 & 2 independently for a sample of 10 studies to check concordance, after which each study will be examined by one reviewer.

**Table 3: Example Data Collection Form**

| Study id | First Author, Year | Study Design | Location | Exposure Definition | Outcome Definition | N subjects | N Exposed | N miscarriage among the exposed | N Stillbirth among the exposed | N miscarriage among the unexposed | N Stillbirth among the unexposed | Statistical Measure and result reported in the paper | Was the study pre or post vaccine rollout? |
|---|---|---|---|---|---|---|---|---|---|---|---|---|---|
| | | | | | | | | | | | | | |

For peer review only

*Assessment of Study Quality*

All included studies will be assessed for bias by reviewers using an adapted version of the National Institute of Clinical Excellence (NICE) Quality appraisal checklist for quantitative studies reporting correlations and associations (Appendix 2)(22). The NICE tool was chosen as it is designed for identifying rigor in observational studies that explore and generate hypotheses about causal relationships and can be used for multiple study designs. The NICE tool consists of five major items: study population and participants, selection and methods, outcomes, analysis, and summary.
Appraisal will be done using an Excel format to allow for easy compilation of responses. Decisions will be discussed any discrepancies resolved. Each study will then be awarded an overall study quality grade for external and internal validity from one of the three categories below which are based on the checklist criteria (Appendix 1).

- ++ All or most of the checklist criteria have been fulfilled, where they have not been fulfilled the conclusions are very unlikely to alter.
- + Some of the checklist criteria have been fulfilled, where they have not been fulfilled, or not adequately described, the conclusions are unlikely to alter.
- – Few or no checklist criteria have been fulfilled and the conclusions are likely or very likely to alter.

Studies deemed to be low quality (category -) will be excluded from any meta-analysis.

*Data Synthesis*

We will use Higgins and Thompson's I- squared statistic to quantify heterogeneity and if $I^2$ is >50% meta-analysis will be conducted in Stata using a Random-effects model (23). Where meta-analysis is attempted funnel plots will be used to assess publication bias (24). Where statistical pooling is not possible, findings will be presented in narrative form using tables to aid in data presentation. If possible, we will conduct subgroup analyses of studies reporting miscarriage and stillbirth separately. We will also look at any potential impact of the widespread use of Covid-19 vaccines by grouping studies into those conducted pre-and post-vaccine role out if possible. We will use 01/03/2021 as the cut-off date for studies considered to be post vaccine roll-out. For studies after this date we will examine the national vaccine rollout programme for the country in which the study was conducted to assess the likelihood that pregnant women within the study would have been vaccinated. We will also consider a subgroup analysis of hospitalised versus non-hospitalised Covid-19 cases if there are enough studies which consider this.

*Patient and Public Involvement*

There will be no patient of public involvement in this project.

## Discussion



For peer review only - http://bmjopen.bmj.com/site/about/guidelines.xhtml

The Covid-19 pandemic has been a challenging time for pregnant women, knowledge on the potential risks of infection to them and their unborn babies is ever evolving. With Covid-19 now circulating widely in many countries and limited risk reduction measures in place it is important to try and fully understand the risks so that pregnant women can be advised appropriately. Reviews and studies to date on whether Covid-19 increases the risk of pregnancy loss have drawn mixed conclusions (2–4,8,13). Covid-19 research is a fast-moving area therefore it is important that reviews are regularly updated. This systematic review aims to provide a comprehensive overview of the latest evidence.

Covid-19 research moves very quickly, and preprint literature has become a key outlet for new research with many researchers opting to make their work available as quickly as possible. Including pre-publications in this review, something which previous reviews have not done, will allow us to obtain as current a picture as possible of all of the evidence. Inclusion of pre-print literature may also help to mitigate any risk of publication bias.

Vaccination against Covid-19 became widely available globally in 2021(25). In the UK pregnant women have been routinely advised to receive COVID-19 vaccination together with the rest of the population, according to their age and underlying health conditions since 16 April 2021(26). The widespread introduction of Covid-19 vaccination may have led to a decrease in potential risk or pregnancy loss. We hope to identify enough studies to allow us to examine separately those which were conducted before and after the vaccination roll out in order to provide an insight into any impact the vaccine may have had.

The results of this review can be used to inform public health messaging for pregnant women around the potential risks of Covid-19 infection. This research will also help to inform any future research studies planned on this question.

## Funding

This work is funded by Wellcome (Senior Research Fellowship for KB, grant number 220283/Z/20/Z). This work forms part of JC's PhD which is funded by The Clinical Practice Research Datalink, a division of the UK Medicines and Healthcare Products Regulatory Agency (Grant number N/A).

## Author Contributions

This protocol was written by JC with KB, RW and MH performing critical review. JC will act as the guarantor of the review.

## Competing Interests

The authors have no competing interests to declare

**Ethics approval**

Not applicable

**Consent for publication**

Not applicable

**Availability of data**

Not applicable



For peer review only - http://bmjopen.bmj.com/site/about/guidelines.xhtml

## Acknowledgments

Not applicable

## References

1. Arthurs AL, Jankovic-Karasoulos T, Roberts CT. COVID-19 in pregnancy: What we know from the first year of the pandemic. Biochim Biophys Acta - Mol Basis Dis. 2021;1867(12).

2. Kazemi SN, Hajikhani B, Didar H, Hosseini SS, Haddadi S, Khalili F, et al. COVID-19 and cause of pregnancy loss during the pandemic: A systematic review. PLoS One [Internet]. 2021;16(8 August):1–10. Available from: http://dx.doi.org/10.1371/journal.pone.0255994

3. Soheili M, Ghobad M, Hamid Reza B, Soheili M, Mahidi Makhtari M, Moradi Y. Clinical manifestation and maternal complications and neonatal outcomes in pregnant women with COVID-19: a comprehensive evidence synthesis and meta-analysis. J Matern Fetal Neonatal Med. 2021;18:1–14.

4. Juan J, Gil MM, Rong Z, Zhang Y, Yang H, Poon LC. Effect of coronavirus disease 2019 (COVID-19) on maternal, perinatal and neonatal outcome: systematic review. Ultrasound Obstet Gynecol. 2020;56(1):15–27.

5. Hazari KS, Abdeldayem R, Paulose L, Kurien N, Almahloul Z, Mohammad H, et al. Covid-19 infection in pregnant women in Dubai: a case-control study. BMC Pregnancy Childbirth [Internet]. 2021;21(1):1–10. Available from: https://doi.org/10.1186/s12884-021-04130-8

6. González Rodríguez L, Oreja Cuesta AB, Pardo Pumar MI, Ferriols-Pérez E, Pedró Carulla R, Bernardo Vega R, et al. SARS-CoV-2 infection in early first-trimester miscarriages: a prospective observational study. Reprod Biomed Online [Internet]. 2022;44(1):127–30. Available from: https://doi.org/10.1016/j.rbmo.2021.09.010

7. Webster WS, Freeman JAD. Prescription drugs and pregnancy. Expert Opin Pharmacother. 2003 Jun;4(6):949–61.

8. Pathirathna ML, Samarasekara BPP, Dasanayake TS, Saravanakumar P, Weerasekara I. Adverse Perinatal Outcomes in COVID-19 Infected Pregnant Women: A Systematic Review and Meta-Analysis. Healthc. 2022;10(2).

9. Wei SQ, Bilodeau-Bertrand M, Liu S, Auger N. The impact of COVID-19 on pregnancy outcomes: A systematic review and meta-analysis. CMAJ. 2021 Apr 19;193(16):E540–8.

10. Sacinti KG, Kalafat E, Sukur YE, Koc A. Increased incidence of first-trimester miscarriage during the COVID-19 pandemic. Ultrasound Obstet Gynecol. 2021;57(6):1013–4.



For peer review only - http://bmjopen.bmj.com/site/about/guidelines.xhtml

11. Francesco D'Antonio, MD; Cihat Sen MDDM. Maternal and perinatal outcomes in high compared to working group on coronavirus disease 2019. 2020;(January).

12. Cosma S, Carosso AR, Cusato J, Borella F, Carosso M, Bovetti M, et al. Coronavirus disease 2019 and first-trimester spontaneous abortion: a case-control study of 225 pregnant patients. Am J Obstet Gynecol [Internet]. 2021;224(4):391.e1-391.e7. Available from: https://doi.org/10.1016/j.ajog.2020.10.005

13. Cavalcante MB, de Melo Bezerra Cavalcante CT, Cavalcante ANM, Sarno M, Barini R, Kwak-Kim J. COVID-19 and miscarriage: From immunopathological mechanisms to actual clinical evidence. J Reprod Immunol [Internet]. 2021;148(September):103382. Available from: https://doi.org/10.1016/j.jri.2021.103382

14. Juan J, Gil MM, Rong Z, Zhang Y, Yang H, Poon LC. Effect of coronavirus disease 2019 (COVID-19) on maternal, perinatal and neonatal outcome: systematic review. Ultrasound Obstet Gynecol. 2020 Jul 1;56(1):15–27.

15. de Medeiros KS, Sarmento ACA, Costa APF, Macêdo LT d. A, da Silva LAS, de Freitas CL, et al. Consequences and implications of the coronavirus disease (COVID-19) on pregnancy and newborns: A comprehensive systematic review and meta-analysis. Int J Gynecol Obstet. 2022 Mar 1;156(3):394–405.

16. Bellos I, Pandita A, Panza R. Maternal and perinatal outcomes in pregnant women infected by SARS-CoV-2: A meta-analysis. Eur J Obstet Gynecol Reprod Biol. 2021 Jan 1;256:194–204.

17. Banaei M, Ghasemi V, Saei Ghare Naz M, Kiani Z, Rashidi-Fakari F, Banaei S, et al. Obstetrics and neonatal outcomes in pregnant women with covid-19: A systematic review. Iran J Public Health. 2020;49:38–47.

18. Shamseer L, Moher D, Clarke M, Ghersi D, Liberati A, Petticrew M, et al. Preferred reporting items for systematic review and meta-analysis protocols (prisma-p) 2015: Elaboration and explanation. BMJ [Internet]. 2015;349(January):1–25. Available from: http://dx.doi.org/doi:10.1136/bmj.g7647

19. Clyne B, Walsh KA, O'Murchu E, Sharp MK, Comber L, O' Brien KK, et al. Using preprints in evidence synthesis: Commentary on experience during the COVID-19 pandemic. J Clin Epidemiol [Internet]. 2021;138:203–10. Available from: https://doi.org/10.1016/j.jclinepi.2021.05.010

20. Kirkham JJ, Penfold NC, Murphy F, Boutron I, Ioannidis JP, Polka J, et al. Systematic examination of preprint platforms for use in the medical and biomedical sciences setting. BMJ Open. 2020;10(12):1–8.

21. Mendeley Ltd. Mendeley Reference Manager [Internet]. 2020. Available from: https://www.mendeley.com



For peer review only - http://bmjopen.bmj.com/site/about/guidelines.xhtml

22. NICE. Methods for the development of NICE public health guidance (third edition ). Natl Inst Heal Clin Excell [Internet]. 2012;(September):284. Available from: https://www.nice.org.uk/process/pmg4/resources/methods-for-the-development-of-nice-public-health-guidance-third-edition-pdf-2007967445701

23. StataCorp. Stata Statistical Software: Release 17. College Station, TX: StataCorp LLC.; 2021.

24. Egger M, Smith GD, Schneider M, Minder C. Bias in meta-analysis detected by a simple, graphical test. Br Med J. 1997;315(7109):629–34.

25. Ritchie H, Mathieu E, Rodés-Guirao L, Appel C, Giattino C, Ortiz-Ospina E, et al. Coronavirus Pandemic (COVID-19) [Internet]. Published online at OurWorldInData.org. 2020. Available from: https://ourworldindata.org/coronavirus

26. UK Health Security Agency. COVID-19: The Green Book chapter 14a. In: The Green Book [Internet]. 2022. Available from: https://www.gov.uk/government/publications/covid-19-the-green-book-chapter-14a%0A

For peer review only

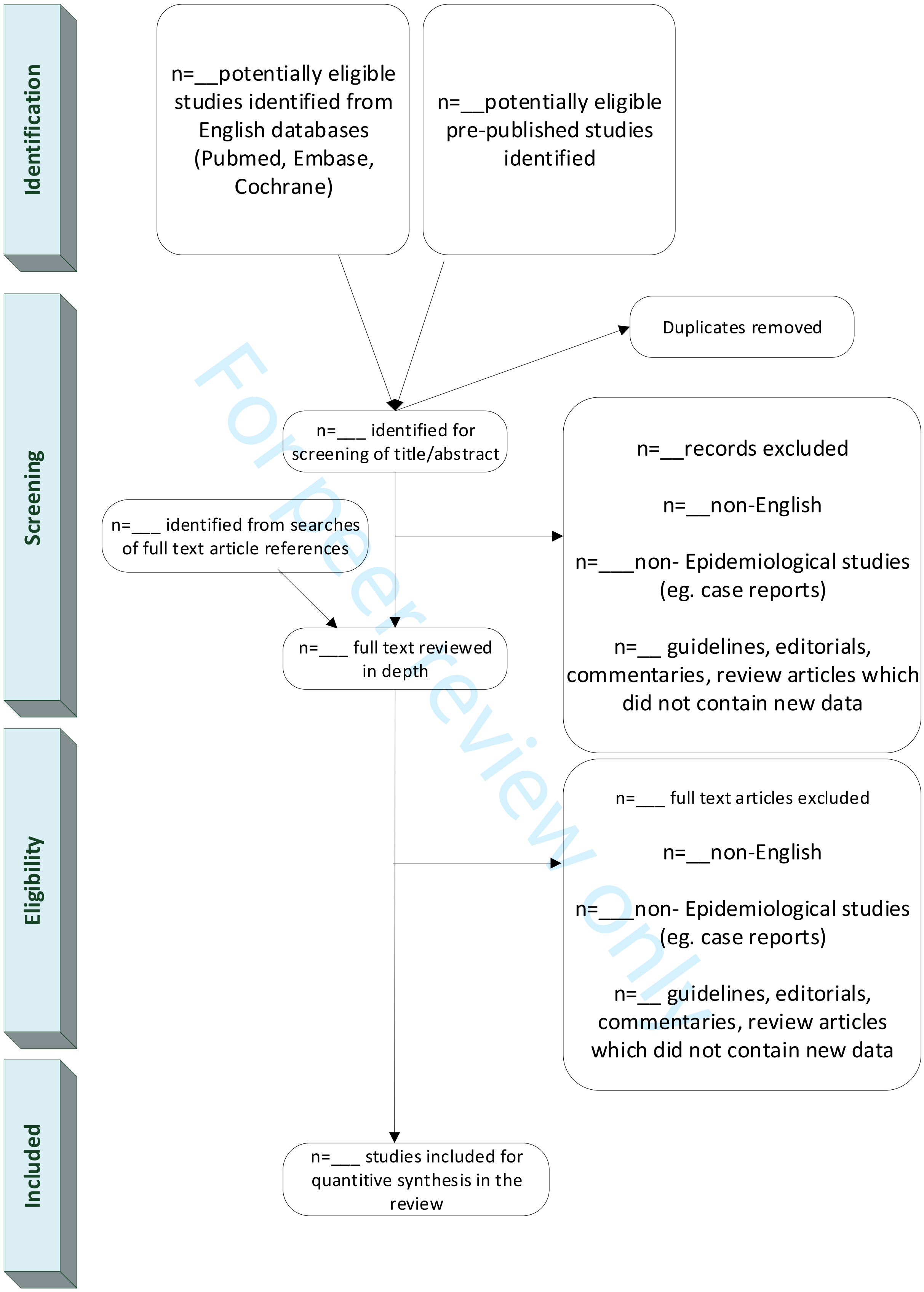


For peer review only

Appendix 1: Prisma-P checklist

**PRISMA-P (Preferred Reporting Items for Systematic review and Meta-Analysis Protocols) 2015 checklist: recommended items to address in a systematic review protocol***

| Section and topic | Item No | Checklist item |
|---|---|---|
| **ADMINISTRATIVE INFORMATION** | | |
| Title: | | |
| Identification | 1a | Identify the report as a protocol of a systematic review |
| Update | 1b | If the protocol is for an update of a previous systematic review, identify as such |
| Registration | 2 | If registered, provide the name of the registry (such as PROSPERO) and registration number |
| Authors: | | |
| Contact | 3a | Provide name, institutional affiliation, e-mail address of all protocol authors; provide physical mailing address of corresponding author |
| Contributions | 3b | Describe contributions of protocol authors and identify the guarantor of the review |
| Amendments | 4 | If the protocol represents an amendment of a previously completed or published protocol, identify as such and list changes; otherwise, state plan for documenting important protocol amendments |
| Support: | | |
| Sources | 5a | Indicate sources of financial or other support for the review |
| Sponsor | 5b | Provide name for the review funder and/or sponsor |
| Role of sponsor or funder | 5c | Describe roles of funder(s), sponsor(s), and/or institution(s), if any, in developing the protocol |
| **INTRODUCTION** | | |
| Rationale | 6 | Describe the rationale for the review in the context of what is already known |
| Objectives | 7 | Provide an explicit statement of the question(s) the review will address with reference to participants, interventions, comparators, and outcomes (PICO) |
| **METHODS** | | |
| Eligibility criteria | 8 | Specify the study characteristics (such as PICO, study design, setting, time frame) and report characteristics (such as years considered, language, publication status) to be used as criteria for eligibility for the review |
| Information sources | 9 | Describe all intended information sources (such as electronic databases, contact with study authors, trial registers or other grey literature sources) with planned dates of coverage |

For peer review only

| | | |
|---|---|---|
| Search strategy | 10 | Present draft of search strategy to be used for at least one electronic database, including planned limits, such that it could be repeated |
| Study records: | | |
| Data management | 11a | Describe the mechanism(s) that will be used to manage records and data throughout the review |
| Selection process | 11b | State the process that will be used for selecting studies (such as two independent reviewers) through each phase of the review (that is, screening, eligibility and inclusion in meta-analysis) |
| Data collection process | 11c | Describe planned method of extracting data from reports (such as piloting forms, done independently, in duplicate), any processes for obtaining and confirming data from investigators |
| Data items | 12 | List and define all variables for which data will be sought (such as PICO items, funding sources), any pre-planned data assumptions and simplifications |
| Outcomes and prioritization | 13 | List and define all outcomes for which data will be sought, including prioritization of main and additional outcomes, with rationale |
| Risk of bias in individual studies | 14 | Describe anticipated methods for assessing risk of bias of individual studies, including whether this will be done at the outcome or study level, or both; state how this information will be used in data synthesis |
| Data synthesis | 15a | Describe criteria under which study data will be quantitatively synthesised |
| | 15b | If data are appropriate for quantitative synthesis, describe planned summary measures, methods of handling data and methods of combining data from studies, including any planned exploration of consistency (such as $I^2$, Kendall's $\tau$) |
| | 15c | Describe any proposed additional analyses (such as sensitivity or subgroup analyses, meta-regression) |
| | 15d | If quantitative synthesis is not appropriate, describe the type of summary planned |
| Meta-bias(es) | 16 | Specify any planned assessment of meta-bias(es) (such as publication bias across studies, selective reporting within studies) |
| Confidence in cumulative evidence | 17 | Describe how the strength of the body of evidence will be assessed (such as GRADE) |

*** It is strongly recommended that this checklist be read in conjunction with the PRISMA-P Explanation and Elaboration (cite when available) for important clarification on the items. Amendments to a review protocol should be tracked and dated.** **The copyright for PRISMA-P (including checklist) is held by the PRISMA-P Group and is distributed under a Creative Commons Attribution Licence 4.0.**

*From: Shamseer L, Moher D, Clarke M, Ghersi D, Liberati A, Petticrew M, Shekelle P, Stewart L, PRISMA-P Group. Preferred reporting items for systematic review and meta-analysis protocols (PRISMA-P) 2015: elaboration and explanation. BMJ. 2015 Jan 2;349(jan02 1):g7647.*

For peer review only

Appendix 2: NICE Quality appraisal checklist for quantitative studies reporting correlations and associations

Checklist items are worded so that 1 of 5 responses is possible: ++ Indicates that for that particular aspect of study design, the study has been designed or conducted in such a way as to minimise the risk of bias. + Indicates that either the answer to the checklist question is not clear from the way the study is reported, or that the study may not have addressed all potential sources of bias for that particular aspect of study design. − Should be reserved for those aspects of the study design in which significant sources of bias may persist. Not reported (NR) Should be reserved for those aspects in which the study under review fails to report how they have (or might have) been considered. Not applicable (NA) Should be reserved for those study design aspects that are not applicable given the study design under review (for example, allocation concealment would not be applicable for case–control studies).

| | | |
|---|---|---|
| **Study identification:** Include full citation details | | |
| **Study design:**<br>• Refer to the glossary of study designs (appendix D) and the algorithm for classifying experimental and observational study designs (appendix E) to best describe the paper's underpinning study design | | |
| **Assessed by:** | | |
| **Section 1: Population** | | |
| **1.1 Is the source population or source area well described?** | ++ | Comments: |

| | | |
|---|---|---|
| • Was the country (e.g. developed or non-developed, type of health care system), setting (primary schools, community centres etc), location (urban, rural), population demographics etc adequately described? | +<br>–<br>NR<br>NA | |
| **1.2 Is the eligible population or area representative of the source population or area?**<br>• Was the recruitment of individuals, clusters or areas well defined (e.g. advertisement, birth register)?<br>• Was the eligible population representative of the source? Were important groups underrepresented? | ++<br>+<br>–<br>NR<br>NA | Comments: |
| **1.3 Do the selected participants or areas represent the eligible population or area?**<br>• Was the method of selection of participants from the eligible population well described?<br>• What % of selected individuals or clusters agreed to participate? Were there any sources of bias? | ++<br>+<br>–<br>NR<br>NA | Comments: |

| | | |
|---|---|---|
| • Were the inclusion or exclusion criteria explicit and appropriate? | | |
| **Section 2: Method of selection of exposure (or comparison) group** | | |
| **2.1 Selection of exposure (and comparison) group. How was selection bias minimised?**<br>• How was selection bias minimised? | ++<br>+<br>−<br>NR<br>NA | Comments: |
| **2.2 Was the selection of explanatory variables based on a sound theoretical basis?**<br>• How sound was the theoretical basis for selecting the explanatory variables? | ++<br>+<br>−<br>NR<br>NA | Comments: |
| **2.3 How well were likely confounding factors identified and controlled?** | ++ | Comments: |

| | | |
|---|---|---|
| • Were there likely to be other confounding factors not considered or appropriately adjusted for?<br>• Was this sufficient to cause important bias? | +<br>−<br>NR<br>NA | |
| **2.4 Is the setting applicable to the UK?**<br>• Did the setting differ significantly from the UK? | ++<br>+<br>−<br>NR<br>NA | Comments: |
| **Section 3: Outcomes** | | |
| **3.1 Were the outcome measures and procedures reliable?**<br>• Were outcome measures subjective or objective (e.g. biochemically validated nicotine levels ++ vs self-reported smoking −)? | ++<br>+<br>− | Comments: |

| | | |
|---|---|---|
| • How reliable were outcome measures (e.g. inter- or intra-rater reliability scores)?<br>• Was there any indication that measures had been validated (e.g. validated against a gold standard measure or assessed for content validity)? | NR<br>NA | |
| **3.2 Were the outcome measurements complete?**<br>• Were all or most of the study participants who met the defined study outcome definitions likely to have been identified? | ++<br>+<br>−<br>NR<br>NA | Comments: |
| **3.3 Were all the important outcomes assessed?**<br>• Were all the important benefits and harms assessed?<br>• Was it possible to determine the overall balance of benefits and harms of the intervention versus comparison? | ++<br>+<br>−<br>NR<br>NA | Comments: |

| | | |
|---|---|---|
| **3.4 Was there a similar follow-up time in exposure and comparison groups?**<br>• If groups are followed for different lengths of time, then more events are likely to occur in the group followed-up for longer distorting the comparison.<br>• Analyses can be adjusted to allow for differences in length of follow-up (e.g. using person-years). | ++<br>+<br>−<br>NR<br>NA | Comments: |
| **3.5 Was follow-up time meaningful?**<br>• Was follow-up long enough to assess long-term benefits and harms?<br>• Was it too long, e.g. participants lost to follow-up? | ++<br>+<br>−<br>NR<br>NA | Comments: |
| **Section 4: Analyses** | | |
| **4.1 Was the study sufficiently powered to detect an intervention effect (if one exists)?** | ++<br>+ | Comments: |

| | | |
|---|---|---|
| • A power of 0.8 (i.e. it is likely to see an effect of a given size if one exists, 80% of the time) is the conventionally accepted standard.<br>• Is a power calculation presented? If not, what is the expected effect size? Is the sample size adequate? | –<br>NR<br>NA | |
| **4.2 Were multiple explanatory variables considered in the analyses?**<br>• Were there sufficient explanatory variables considered in the analysis? | ++<br>+<br>–<br>NR<br>NA | Comments: |
| **4.3 Were the analytical methods appropriate?**<br>• Were important differences in follow-up time and likely confounders adjusted for? | ++<br>+<br>–<br>NR<br>NA | Comments: |

| | | |
|---|---|---|
| **4.6 Was the precision of association given or calculable? Is association meaningful?**<br>• Were confidence intervals or p values for effect estimates given or possible to calculate?<br>• Were CIs wide or were they sufficiently precise to aid decision-making? If precision is lacking, is this because the study is under-powered? | ++<br>+<br>−<br>NR<br>NA | Comments: |
| **Section 5: Summary** | | |
| **5.1 Are the study results internally valid (i.e. unbiased)?**<br>• How well did the study minimise sources of bias (i.e. adjusting for potential confounders)?<br>• Were there significant flaws in the study design? | ++<br>+<br>− | Comments: |
| **5.2 Are the findings generalisable to the source population (i.e. externally valid)?**<br>• Are there sufficient details given about the study to determine if the findings are generalisable to the source population? | ++<br>+<br>− | Comments: |

| | | |
|---|---|---|
| • Consider: participants, interventions and comparisons, outcomes, resource and policy implications. | | |

For peer review only

# Reporting checklist for protocol of a systematic review and meta analysis.

Based on the PRISMA-P guidelines.

## Instructions to authors

Complete this checklist by entering the page numbers from your manuscript where readers will find each of the items listed below.

Your article may not currently address all the items on the checklist. Please modify your text to include the missing information. If you are certain that an item does not apply, please write "n/a" and provide a short explanation.

Upload your completed checklist as an extra file when you submit to a journal.

In your methods section, say that you used the PRISMA-Preporting guidelines, and cite them as:

Moher D, Shamseer L, Clarke M, Ghersi D, Liberati A, Petticrew M, Shekelle P, Stewart LA. Preferred Reporting Items for Systematic Review and Meta-Analysis Protocols (PRISMA-P) 2015 statement. Syst Rev. 2015;4(1):1.

| | | Reporting Item | Page Number |
|---|---|---|---|
| **Title** | | | |
| Identification | #1a | Identify the report as a protocol of a systematic review | 1 |
| Update | #1b | If the protocol is for an update of a previous systematic review, identify as such | n/a |
| **Registration** | | | |
| | #2 | If registered, provide the name of the registry (such as PROSPERO) and registration number | 1 |
| **Authors** | | | |
| Contact | #3a | Provide name, institutional affiliation, e-mail address of all protocol authors; provide physical mailing address of corresponding author | 1 |
| Contribution | #3b | Describe contributions of protocol authors and identify the guarantor of the review | 8 |

For peer review only

| | | | |
|---|---|---|---|
| **Amendments** | | | |
| | #4 | If the protocol represents an amendment of a previously completed or published protocol, identify as such and list changes; otherwise, state plan for documenting important protocol amendments | n/a |
| **Support** | | | |
| Sources | #5a | Indicate sources of financial or other support for the review | 8 |
| Sponsor | #5b | Provide name for the review funder and / or sponsor | 8 |
| Role of sponsor or funder | #5c | Describe roles of funder(s), sponsor(s), and / or institution(s), if any, in developing the protocol | n/a |
| **Introduction** | | | |
| Rationale | #6 | Describe the rationale for the review in the context of what is already known | 2 |
| Objectives | #7 | Provide an explicit statement of the question(s) the review will address with reference to participants, interventions, comparators, and outcomes (PICO) | 2 |
| **Methods** | | | |
| Eligibility criteria | #8 | Specify the study characteristics (such as PICO, study design, setting, time frame) and report characteristics (such as years considered, language, publication status) to be used as criteria for eligibility for the review | 2-3 |
| Information sources | #9 | Describe all intended information sources (such as electronic databases, contact with study authors, trial registers or other grey literature sources) with planned dates of coverage | 3-4 |
| Search strategy | #10 | Present draft of search strategy to be used for at least one electronic database, including planned limits, such that it could be repeated | 3-4 |
| Study records - data management | #11a | Describe the mechanism(s) that will be used to manage records and data throughout the review | 4-5 |
| Study records - selection process | #11b | State the process that will be used for selecting studies (such as two independent reviewers) through each phase of the review (that is, screening, eligibility and inclusion in meta-analysis) | 5 |
| Study records - data | #11c | Describe planned method of extracting data from reports (such as | 6 |

For peer review only

| | | | |
|---|---|---|---|
| collection process | | piloting forms, done independently, in duplicate), any processes for obtaining and confirming data from investigators | |
| Data items | #12 | List and define all variables for which data will be sought (such as PICO items, funding sources), any pre-planned data assumptions and simplifications | 6 |
| Outcomes and prioritization | #13 | List and define all outcomes for which data will be sought, including prioritization of main and additional outcomes, with rationale | 2 |
| Risk of bias in individual studies | #14 | Describe anticipated methods for assessing risk of bias of individual studies, including whether this will be done at the outcome or study level, or both; state how this information will be used in data synthesis | 7 |
| Data synthesis | #15a | Describe criteria under which study data will be quantitatively synthesised | 7 |
| Data synthesis | #15b | If data are appropriate for quantitative synthesis, describe planned summary measures, methods of handling data and methods of combining data from studies, including any planned exploration of consistency (such as I2, Kendall's τ) | 7 |
| Data synthesis | #15c | Describe any proposed additional analyses (such as sensitivity or subgroup analyses, meta-regression) | 7 |
| Data synthesis | #15d | If quantitative synthesis is not appropriate, describe the type of summary planned | 7 |
| Meta-bias(es) | #16 | Specify any planned assessment of meta-bias(es) (such as publication bias across studies, selective reporting within studies) | 7 |
| Confidence in cumulative evidence | #17 | Describe how the strength of the body of evidence will be assessed (such as GRADE) | 7 |

The PRISMA-P elaboration and explanation paper is distributed under the terms of the Creative Commons Attribution License CC-BY. This checklist was completed on 09. June 2022 using https://www.goodreports.org/, a tool made by the EQUATOR Network in collaboration with Penelope.ai

For peer review only
